# The Engagement Between MDSCs and Metastases: Partners in Crime

**DOI:** 10.3389/fonc.2020.00165

**Published:** 2020-02-18

**Authors:** Rosalinda Trovato, Stefania Canè, Varvara Petrova, Silvia Sartoris, Stefano Ugel, Francesco De Sanctis

**Affiliations:** Section of Immunology, Department of Medicine, University of Verona, Verona, Italy

**Keywords:** MDSCs (myeloid-derived suppressor cells), immunosuppression, metastases, metastatic process, pre-metastatic niche

## Abstract

Tumor metastases represent the major cause of cancer-related mortality, confirming the urgent need to identify key molecular pathways and cell-associated networks during the early phases of the metastatic process to develop new strategies to either prevent or control distal cancer spread. Several data revealed the ability of cancer cells to establish a favorable microenvironment, before their arrival in distant organs, by manipulating the cell composition and function of the new host tissue where cancer cells can survive and outgrow. This predetermined environment is termed “pre-metastatic niche” (pMN). pMN development requires that tumor-derived soluble factors, like cytokines, growth-factors and extracellular vesicles, genetically and epigenetically re-program not only resident cells (i.e., fibroblasts) but also non-resident cells such as bone marrow-derived cells. Indeed, by promoting an “emergency” myelopoiesis, cancer cells switch the steady state production of blood cells toward the generation of pro-tumor circulating myeloid cells defined as myeloid-derived suppressor cells (MDSCs) able to sustain tumor growth and dissemination. MDSCs are a heterogeneous subset of myeloid cells with immunosuppressive properties that sustain metastatic process. In this review, we discuss current understandings of how MDSCs shape and promote metastatic dissemination acting in each fundamental steps of cancer progression from primary tumor to metastatic disease.

## Introduction

At steady-state, peripheral myeloid cells, such as monocytes and neutrophils, are constantly replenished by new cells originated from hematopoietic stem and progenitor cells (HSPCs) located in the bone marrow (BM) following tightly regulated biological processes (1–4). This constant turnover, termed myelopoiesis, has a profound impact on the BM activity, since approximately hundreds of millions of myeloid cells are generated everyday (5). These myeloid effector cells control localized infections preventing bacterial dissemination without altering the physiological BM cellular output. In contrast, in the presence of a severe infection, injury and stress, the release of inflammatory cytokines and chemokines as well as the activation of damage- or pathogen-associated molecular patterns (DAMPs and PAMPs, respectively) can systemically alter the development of myeloid cells favoring the generation of a large amount of *de novo* BM-derived cells. This abnormal process is termed as “emergency” myelopoiesis (6, 7) and, in clinical settings, it is characterized by an increased number of neutrophils (neutrophilia) and the presence of circulating immature myeloid precursors (“left shift”). The overall goal of this time-regulated process is the continuous replenishment of myeloid cells that are consumed in the battle against pathogens until the return to a steady-state condition. However, this flexible and powerful system can be corrupted by cancer cells to establish a stable inflammation state that sustains a long-lasting altered myelopoiesis (8). For this reason, tumor-promoting inflammation has been listed among tumor hallmarks (9). Indeed, by releasing several tumor-derived soluble factors (TDSFs), such as growth factors [i.e., granulocyte colony-stimulating factor (G-CSF) and granulocyte macrophage-colony stimulating factor (GM-CSF)], pro-inflammatory cytokines (i.e., interleukin (IL)-6, IL-1β and tumor-necrosis factor (TNF)-α) (10–12), as well as by tumor-derived exosomes (TEXs) shedding (13), cancer cells can orchestrate and maintain this abnormal hematopoietic response. Accordingly, it has been recently demonstrated that lethally irradiated mice transplanted with TEX-educated BM cells possess greater number of BM-derived cells inside the primary tumor mass as well as a greater metastatic burden than controls, suggesting the ability of TEXs to manipulate the hematopoietic cell proliferation and lineage differentiation programs (13). Similarly, several reports highlight an impairment of the HSPC hierarchy mediated by TDSFs which reduce the number of quiescent pluripotent stem cells, through the activation of alternative signaling pathways, promoting the accumulation of high number of immature and mature cells in the BM and in the periphery of tumor-bearing hosts (14–18). In the light of these premises, the increased neutrophil-to-lymphocyte ratio (NLR), that is a simple clinical parameter to evaluate systemic inflammation, has been confirmed as a suitable prognostic and predictive value for patient outcome in different cancer settings (19, 20). This close relationship between BM-derived immune cells and cancer cells raises several basic questions: why do cancer cells orchestrate and promote the alteration of BM-derived cell generation? Which is the result of tumor-driven myelopoiesis? Which is the impact of tumor-educated myeloid cells on tumor progression? Apparently, the final goal of cancer cells is to generate myeloid partners that fuel and sustain its growth and spreading and, among them, myeloid-derived suppressor cells represent the most attractive candidate.

## MDSC: A Tumor-Induced Myeloid Cell Subset

Myeloid-derived suppressor cells (MDSCs) are a heterogeneous myeloid cell population characterized by immune regulatory properties (21, 22). The differentiation and accumulation of MDSCs in human beings depends on pathological conditions such as cancer (23), infection (24), autoimmunity (25) and transplantation (26) but occurs during physiological processes such as aging (27) and pregnancy (28). MDSCs can be divided at least in three main subgroups according to the expression of selective surface markers: monocytic MDSC (M-MDSCs), that are characterized as CD11b^+^Ly6C^+^Ly6G^−^ cells in mouse and CD11b^+^CD14^+^CD15^−^HLA-DR^low/−^CD124^+^ cells in human; polymorphonuclear-MDSC (PMN-MDSCs), that are identified as CD11b^+^Ly6C^−^Ly6G^+^ cells in tumor-bearing mice and CD11b^+^CD14^−^CD15^+^HLA-DR^low/−^CD124^+^ cells in cancer patients (when the analysis is performed in low density mononuclear cell fraction); finally, the last MDSC subset is composed by “early immature” MDSCs (eMDSCs) defined as CD11b^+^Gr1^+^CCR2^+^Sca1^+^CD31^+^ cells in mouse and Lin^−^CD11b^+^CD34^+^CD33^+^CD117^+^HLA-DR^low/−^ cells in human (8, 21, 29). Since MDSCs share some phenotypic and morphologic features with the normal counterpart (i.e., neutrophils and monocytes) (22), their unequivocal identification needs to be proved by functional *in vitro* assays (22, 30). In fact, we recently demonstrated that, immunosuppressive monocytes isolated from the blood of pancreatic ductal adenocarcinoma (PDAC) patients resembling M-MDSCs, were not distinguishable from normal monocytes by the expression of a specific surface markers but, instead, by cytological features (i.e., smaller size, presence of granules), immune suppressive properties and molecular signatures (31), suggesting the existence of a high heterogeneity and complexity among the M-MDSC subsets. Similarly, the discrimination between PMNs and PMN-MDSCs based on differential expression level of surface markers has recently generated a lot of controversies [as discussed in (32, 33)] suggesting that only a complementary analysis of genomic, proteomic, and biochemical characteristics would precisely pinpoint the target cell population. Even if several phenotypic markers have been proposed to be exclusive of MDSCs [i.e., CD38 (34), TNFR (35)], so far none of them has been proved has unequivocal target for MDSC [as recently reviewed in (22, 36)]. Only the expression of the lectin-type oxidized LDL receptor 1 (LOX-1) was reported to be exclusive of PMN-MDSCs (37), but more studies in different patient cohorts need to be done.

In general, M-MDSCs are more immunosuppressive than PMN-MDSCs on a per cell basis both in tumor-bearing mice (15, 38) and cancer patients (31). Moreover, M-MDSCs exhibit longer half-life and more pronounced cell plasticity compared to PMN-MDSCs since they are able to differentiate into tumor-associated macrophages (TAMs) (39), as well as they can act as “precursors” to maintain circulating PMN-MDSCs level (38). Indeed, in tumor-bearing but not in tumor-free mice, M-MDSCs acquire PMN-MDSC-associated features through an epigenetic mechanism based on downregulation of retinoblastoma protein expression by histone deacetylase enzymes (40). Notably, M- and PMN-MDSCs display also distinctive cell-death programs. In fact, the anti-apoptotic molecules c-FLIP (cellular FLICE [FADD-like IL-1β-converting enzyme]-inhibitory protein) and MCL-1 are essential for the development of M-MDSCs and PMN-MDSCs, respectively (41). Interestingly, we recently demonstrated that c-FLIP plays an essential role on re-programming exclusively monocytes into MDSCs without affecting cell survival since this mechanism does not affect neutrophils conversion into PMN-MDSCs. In addition, we unveiled c-FLIP as a new regulator of nuclear factor kappa-light-chain-enhancer of activated B cells (NF-κB) signaling by interaction with the p50 subunit in the nucleus therefore promoting the aberrant transcription of several immunosuppression-related genes (42). Nowadays, in the single-cell omics era, it is quite accepted that M-MDSCs and PMN-MDSCs represent the two major extremes of a continuous spectrum of myeloid cells differentiation induced by tumor and only the application of high resolution transcriptome technologies will shed light on the ontogeny of the complex and variegated world of MDSC. In line, recent publications clearly showed that MDSCs originate “unexpected” cell subsets like dendritic cells (DCs) (43) or fibroblasts (44) in response to diverse microenvironmental stimuli.

The MDSC plasticity and functions are strictly guided by the activation of precise signaling pathways [extensively reviewed in (8, 45)] preferentially driven by c/EBPβ (CCAAT/enhancer-binding protein) (16), STAT3 (signal transducer and activator of transcription 3) (31, 46) and NF-κB (42, 47) transcriptional factors. c/EBPβ is the master regulator of “emergency” myelopoiesis and its critical role on MDSC biology was proved using myeloid-restricted c/EBPβ-deficient mice engrafted with different tumor models in which the ontogeny and MDSC-associated immunosuppression were completely abrogated (16). Recently, Strauss and collaborators demonstrated that c/EBPβ-guided myelopoiesis can be sustained by myeloid-specific expression of the retinoic-acid related orphan receptor (RORC1/RORγ) (17) promoting MDSC and TAM expansion. Furthermore, MDSC generation and accumulation in tumor-bearing mice can also be driven by the c/EBP homologous protein (CHOP)-mediated signaling (48). CHOP is the master sensor of endoplasmic reticulum (ER) stress such as low pH, high levels of reactive oxygen species (ROS, i.e., H_2_O_2_), nitric-oxide (NO), hypoxia, nutrient deprivation, etc. (49). Interestingly, ER stress-inducers like thapsigargin promote *in vitro* differentiation of human neutrophils to PMN-MDSCs (37). Similarly, GCN2 (general control non-derepressible 2), that is a master environmental sensor able to control transcription and translation in response to nutrient availability, was reported to drive and sustain immunosuppressive functions of MDSCs in tumor microenvironment (50). STAT3 plays a central role in regulating both the expansion and the tolerogenic effects of MDSCs. STAT3 preserves MDSC survival by upregulating B-cell lymphoma XL (Bcl-X_L_), c-Myc, Cyclin D1 and survivin (51, 52), and by blocking myeloid cell differentiation through the downregulation of interferon regulatory factor (IRF) 8 expression (53). STAT3 controls many MDSC-released mediators (cytokines, growth factors, enzymes) that promotes pro-tumor effects. In particular activated STAT3 triggers on one hand the production of pro-inflammatory proteins, like S100A8/A9 (54) that interfere with DC differentiation and sustain ROS generation (55); on the other hand by binding to the arginase 1 (*ARG1*) promoter, STAT3 favors its aberrant expression (46). Interestingly, we recently demonstrated a unique STAT3-dependent expression of ARG1 in a subset of cancer patient-derived monocytes (31). NF-κB, the master regulator of inflammation, was reported to be involved in MDSC differentiation. Recently, Sangaletti and collaborators demonstrated that impaired translocation of NF-κB p50 protein abolishes the secretion of protein acidic and rich in cysteine (SPARC) and alters MDSC-associated immunosuppression by limiting ROS production. Indeed, restricted p50 translocation into nucleus limits the formation of the immunosuppressive p50:p50 homodimers in favor of the p65:p50 inflammatory heterodimers that sustain an increased release of TNFα in the tumor microenvironment (47). According to this, we demonstrated that, the enhancement of nuclear p50 translocation by c-FLIP promotes acquisition of immunosuppressive function by monocytes (42). Together, these data highlight a pivotal role of p50 on driving MDSC differentiation that needs to be better investigated in the near future.

Classically, MDSC pro-tumor functions are ascribed for the effects on the adaptive immune response. However, recent insights on MDSC field demonstrated that these tumor-educated cells sustain tumor growth by also non-immune processes such as by promoting angiogenesis, maintaining cancer cell-stemness and sustaining the metastatic process. Since metastatic spreading is essentially inefficient whereby the majority of cancer cells cannot rich or seed to distant sites, tumors need to develop strategies to both inhibit immune response and alter tissue framework. Thus, in this context, it is clear that MDSCs represent the best partner for tumor cells since circulating MDSCs can support tumor cell during each step of the metastatic process.

## MDSCs Involvement During Different Stages of Metastatic Process

**Figure 1:**
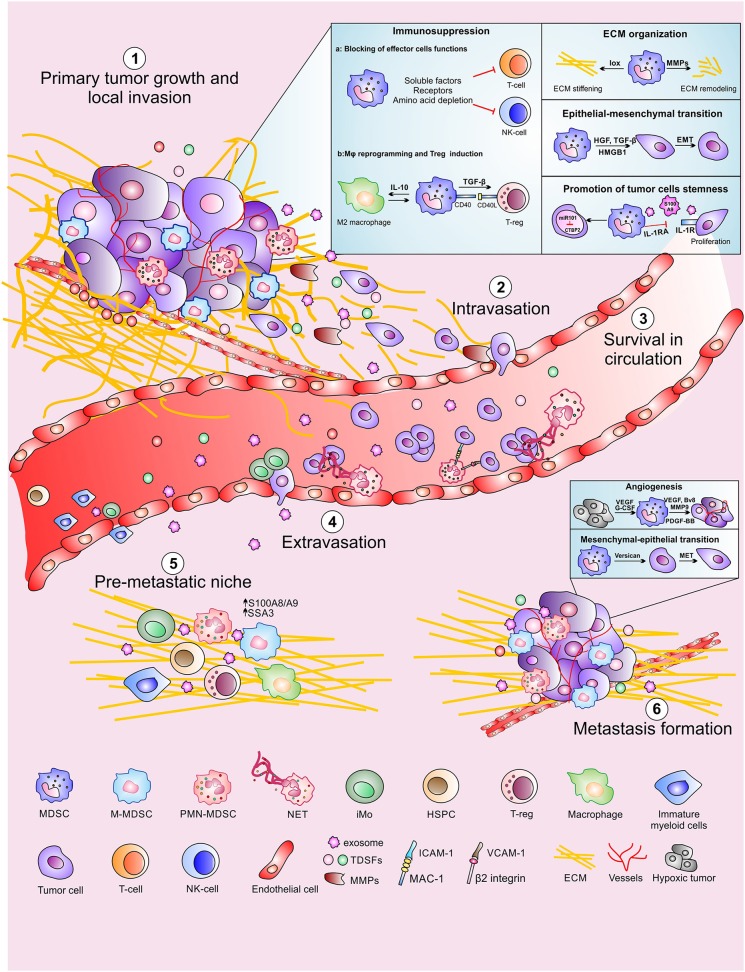
MDSCs contribution to the different steps of the metastatic cascade. MDSCs promote primary tumor growth and local invasion (1) with several mechanisms including suppression of adaptive immune response, ECM reorganization, promotion of epithelial-mesenchymal transition as well as maintaining tumor cells stemness. MDSCs also support distal tumor spread by favoring tumor cells intravasation (2), CTC survival in circulation (3) and CTC extravasation at the metastatic site. Moreover, MDSCs contribute to the formation of the pre-metastatic niche (5) in which CTC can proliferate promoting the metastasis formation (6).

Metastasis is a stepwise process that drives cancer's outgrowth to an organ different from which they originated. Indeed, cancer cells, after acquiring an invasive phenotype by accumulation of genetic and epigenetic aberrations (*primary tumor growth*), can invade the surrounding tissues (*local invasion*) and infiltrate into the blood stream or lymph vessels (*intravasation*) turning into anchorage-independent circulating tumor cells (CTCs). After intravasation, CTCs need to stay alive (*survival in circulation*) until they exit from the circulation (*extravasation*) and adapt themselves to a new tissue (*pre-metastatic niche*) to generate a secondary tumor mass (*metastasis formation*) (56, 57) as depicted in Figure 1. Here we will describe the role of MDSCs on the different steps of the metastatic cascade.

### MDSCs Promote Primary Tumor Growth and Local Invasion

**Figure 2:**
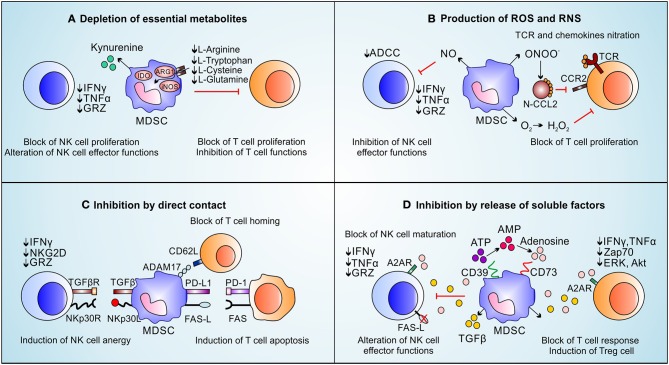
Immune suppressive functions of MDSCs on NK cells and T-cells. MDSCs inhibit immune effector cells by exploting four main mechanisms: **(A)** MDSCs deplete essential metabolites for T lymphocyte fitness (i.e., L-arginine, L-tryptophan, L-cysteine, and L-glutamine) which induce T cell proliferation arrest. L-arginine depletion, promoted by ARG1 activity, induces the loss of the CD3ζ chain affecting T cells response to various stimuli. The kynurenines, produced during L-tryptophan catabolism by IDO, block NK cells proliferation, activation and functions. **(B)** MDSCs produce ROS and RNS. The release of NO inhibits FC-receptor-mediated ADCC in NK cells and reduces their effector functions. High levels of ROS downregulate CD3ζ chain expression and reduce cytokine secretion on T cells. RNS also block T cells recruitment and proliferation by nitration/nitrosylation of chemokines (CCL2, CCL5, CCL21, CXCL12) and TCR. **(C)** MDSCs suppress NK cells and T cells by direct contact. MDSCs, through membrane-bound TGF-β and NKp30L, promote NK cell anergy. MDSCs block the T cell homing through CD62L/ADAM17 interaction; moreover, MDSCs express PD-L1 and FAS-L, which binding their receptors on T cells, promote T-cell apoptosis. **(D)** MDSCs induce immune suppression through the release of soluble factors: MDSCs present high levels of CD39 and CD73 able to transform ATP in adenosine. High amount of adenosine affect NK maturation as well as NK and T-cell effector functions. Moreover, by TGF-β release, MDSCs induce Treg cells and reduce IFNy, TNFα, and GRZ release by NK cells.

MDSCs promote primary tumor progression by both immunological and non-immunological mechanisms (8, 29). The immunological pro-tumor functions of MDSCs is exploited by suppressing both innate and adaptive immune responses. Indeed, MDSCs support the generation of a hostile tumor microenvironment by producing metabolites and soluble factors, as well as by expressing membrane-bound proteins which interfere with effector T cell function and fitness (58) or by promoting the generation of Foxp3 (forkhead box P3)-expressing immunosuppressive B regulatory (Breg) (59) and T regulatory (Treg) lymphocytes (60) as summarized in Figure 2. In this context, the depletion of essential aminoacids, such as arginine, tryptophan, cysteine and glutamine represents a key strategy (61). MDSCs co-express ARG1 and inducible nitric oxide synthase (iNOS, NOS2), which compete for the same substrate, arginine, in order to produce ornithine and urea or NO and citrulline, respectively (62). Arginine depletion reduces the expression of cyclin D3, cyclin dependent kinase 4 (cdk4), and E2F1 transcription factor in T cells favoring their cell cycle arrest in G0-G1 phase and anergy (63). Moreover, the reduced arginine availability affects the TCR ζ-chain expression in T lymphocytes, limiting thus their activation, proliferation and cytokine production (64). Interestingly, some polyamines (i.e., spermidine) produced by ARG1-dependent pathway activate indoleamine 2,3-dioxygenase 1 (IDO1) expression and signaling, thus constituting keys elements for the crosstalk between these two enzymes (65, 66). IDO1 is the most up-regulated tryptophan (Trp)-catabolizing enzyme in tumor-infiltrating MDSCs and tolerigenic DCs (67). IDO1 catabolizes Trp into NAD^+^ (nicotinamide adenine dinucleotide), an essential pyridinenucleotide that orchestrates several cell-associated biological processes, through the production of kynurenines (68). The latter, by binding to the aryl-hydrocarbon receptor, promote both T lymphocytes and antigen presenting cells (APCs) switch into Tregs and IDO1-expressing tolerogenic DCs, respectively (69). Similarly to the effect of arginine depletion, Trp consumption was shown to promote the down-regulation of TCR ζ-chain favoring T cell anergy (70). Moreover, kynurenine accumulation was reported to inhibit NK cell function and proliferation (71). When the physiological amount of arginine in the tumor microenvironment drastically decreases, iNOS generates superoxide anion (${\text{O}}_{2}^{-}$) by a biochemical process called “uncopling reaction” (72). This unstable agent rapidly produces aberrant reactive nitrogen species (RNS) such as peroxinitrites (ONOO^−^). RNS promote protein post-translational modifications (PTMs) which irreversibly alter protein functions. PTMs finely tune the immune response in the tumor microenvironment by affecting different T cell-dependent signaling pathways and biological processes. Indeed, PTMs modifying both chemokines (i.e., C-C chemokine ligand 2 and 5, CCL2 and CCL5) and immune receptors (i.e., peptide-MHC complex, pMHC), damp both T lymphocyte migration toward primary tumor site (73) and T cell activation and persistence (74), respectively. In fact, tumor-bearing mice treated with AT38 ([3-(aminocarbonyl) furoxan-4-yl] methyl salicylate), an ARG1 and iNOS transcriptional inhibitor, displayed a strong reduction of nitro-tyrosine (NTy)-based PTMs in tumor microenvironment favoring T-cell infiltration inside the tumor and improving anti-tumor immunotherapy (73). Similarly to RNS, high amounts of ROS and NO in tumor microenvironment reduce also antigen specific T cell response by affecting TCR-associated (75, 76) or IL2R-dependent (77) signaling pathways. Moreover, MDSC-released NO reduces Fc receptor-mediated antibody-dependent cellular cytotoxicity (ADCC) of NK cells and alters their effector functions inhibiting IFNγ and TNFα secretion (78). The production of ROS by MDSCs preferentially depends on NADPH oxidases (NOX family) (79) and promotes the activation of several inflammatory target genes such as cyclooxygenase-2 (COX-2) (80). Notably, the inhibition of ROS generation through the addition of either catalase, an enzyme that detoxifies hydrogen peroxide, or Celecoxib, a COX-2 inhibitor, effectively impaired the MDSC immunosuppressive function *in vitro* (81, 82). Recently, we demonstrated that ARG1 has a hierarchical negative function as compared to iNOS in establishing an immunosuppressive tumor microenvironment since tumor-infiltrating, iNOS-expressing myeloid cells (defined as Tip-DC) efficiently sustain anti-tumor T cell activities on debulking tumor mass (83). The pro-tumor role of ARG1 was partially confirmed by clinical evidences. Indeed, the frequency of ARG1-expressing MDSCs significantly discriminate PDAC metastatic patients suggesting that these cells have a pro-metastatic potential (31), as well as the reduction of ARG1^+^ cells in melanoma patients after Ipilimumab-based treatment highlights that the therapeutic efficacy of this immune-based treatment might involve a systemic effect on MDSC accumulation (84). Indeed, a contraction of MDSCs in a Durvalumab responder patient was also reported in lung adenocarcinoma setting (85), suggesting that MDSC enumeration might be a useful biomarker to stratify immunotherapy-undergoing patients. All these evidences will be validated in a large number of immunotherapy-based clinical trials in the next years.

Another MDSC-associated strategy to inhibit T cells depends on the release of soluble factors, especially anti-inflammatory cytokines. Tumor growth factor (TGF-)-β for instance suppresses CD4-expressing T helper (Th) lymphocyte differentiation toward Th1 and Th2 phenotype by altering T-bet and GATA3 expression (86–88). Moreover, TGF-β in association with either IL-10 or specific cell-to-cell contacts [i.e., CD40/CD40L (89)] promotes not only the conversion of naïve T cells into Tregs (90) but also the macrophage polarization toward M2 status through an autocrine positive loop (91). Furthermore, MDCSs induce NK cells anergy through the membrane-bound TGF-β (92). Interestingly, TGF-β-produced by MDSCs promotes the expression of programmed cell death-1 (PD1) in T cells (93). Similarly, MDSCs can hinder T cell fitness and function by directly binding FASL and PDL1 with respective death receptor ligands expressed on T cell surface (94, 95). In this context, for example, the β2 adrenergic receptor triggering induces STAT3-mediated up-regulation of death receptor ligands in MDSCs, potentiating their T cell dysfunction abilities (95). Notably, the transcriptional expression of PDL1 in MDSCs is strictly controlled by TDSFs such as vascular endothelial growth factor (VEGF) and macrophages colony-stimulating factor (M-CSF) (96) as well as by hypoxia-inducible factor (HIF-)1α signaling pathway (97). Interestingly, the activation of HIF-1α in MDSCs favors also the expression of ectonucleoside triphosphate disphosphohydrolase 2 (NTPDase2/CD39L1), an ectoenzyme that controls MDSCs accumulation (98) as well as the expression of (NTPDase1/CD39) and ecto-5′-nucleotidase (Ecto5'NTase/CD73) directly involved in the generation of extracellular adenosine (99), known inhibitor of T cell activation by Zap70-, ERK- and Akt-associated pathway blockade (100) and NK effector functions reducing granzyme, IFNγ and TNFα release (101).

By exploring all these multiple immune-related mechanisms, MDSCs generate a physical and chemical shield against T lymphocytes that protects cancer cells. However, MDSCs are also actively involved in non-immunological processes that sustain tumor local invasion by altering directly tumor cells or the tissues around. In fact, the uncontrolled tumor growth implies profound changes in the adhesion and migratory properties of the tumor cells, which favor cellular dissociation and migration to adjacent tissues, as well as key alterations of tissue framework such as extracellular matrix (ECM) composition. To sustain tumor progression, MDSCs can drive tumor cells to lose epithelial features and the gain of a mesenchymal phenotype, a process known as epithelial-to-mesenchymal transition (EMT), through the release of soluble factors (102). In melanoma bearing mice, in fact, PMN-MDSCs induce EMT by releasing TGF-β and hepatocyte growth factor (HGF) (103); moreover EMT was finely tuned by MDSC-secreted factors such as TGF-β release in combination with high amount of NO in nasopharyngeal carcinoma (104). Moreover, both MDSCs and tumor cells secreted high-mobility group box-1 (HMGB1), a damage-associated molecular pattern protein whose signaling trough both Toll-like receptors (TLRs) and receptor for advanced glycation end products (RAGE), activates EMT-inducing transcription factors (i.e., Snail and NF-κB) and up-regulates matrix metalloproteinase-7 (MMP7) (105). However HMGB1 is a pleiotropic molecule that shows pro-tumor and tumor-restricting actions in a context specific manner (106, 107). MDSCs are also capable to preserve cancer cell intrinsic properties such as cellular stemness. For instance, the direct contact between MDCSs and ovarian cancer cells induced a stem-like phenotype in tumor cells and enhanced their ability to metastasize *in vivo*. This effect is mediated by microRNA-101 up-regulation in neoplastic cells and the subsequent inhibition of the co-repressor gene C-terminal binding protein-2 (CtBP2), which modulates the expression of stem cell genes (108). PMN-MDSCs can also block senescence in cancer cells by promoting their growth through the release of IL-1 receptor antagonist (109) or S100A9-expressing exosomes (110). Finally, in both mouse and human pancreatic tumors, M-MDSCs induce the expansion of aldehyde dehydrogenase-1 (ALDH1)–expressing cancer stem cells that are characterized by higher metastatic potential (111).

Furthermore, MDSCs can actively support tumor progression by acting on the physical framework of local tissue. Indeed, MDSCs support tumor invasion by ECM remodeling and rearrangement of the epithelial basement membrane as well as by modifying matrix stiffness (112). ECM is composed of different macromolecules including collagens, fibronectin, laminin, proteoglycans and polysaccharides and regulates many cellular functions such as cell adhesion, proliferation and migration (113). This complex structure can be remodeled by both tumor cells and MDSCs that release high amounts of degrading enzymes such as MMPs and cathepsins (114, 115). Indeed, MDSCs produce high levels of MMPs, including MMP2, MMP8, MMP9, MMP13, and MMP14, which by digesting ECM allow tumor cells migration (116). Furthermore, the remodeling of ECM increases the bioavailability of matrix-bound factors such as TGF-β and VEGF which further prompt tumor cell invasiveness and angiogenesis (114, 117). Notably, MDSC-released TGF-β induces the production of lysyl oxidase (LOX), which cross-links collagen fibers and other ECM components. LOX overexpression in breast cancer increases ECM stiffness which could promote tumor cell invasion and intravasation by enhancing integrin-dependent mechanotransduction (118, 119). Moreover, ECM structure and composition can influence many aspects of MDSC behaviors, including infiltration, differentiation, and function generating a sort of vicious cycle that favors tumor growth and dissemination (113). In light of these evidences, it is not surprising that SPARC, a matricellular protein produced by tumor cells, promotes the expansion and recruitment of MDSCs (120). In turn, the ablation of SPARC in PMN-MDSCs reduces their suppressive activity and their capacity to sustain EMT and tumor growth (47). Similarly, silencing osteopotin (OPN), a matrix protein, in 4T1 breast cancer cells prevents metastasis development by affecting M-MDSC suppressive activity but not their recruitment at the metastatic site (121). The complement system plays also a pivotal role in promoting the metastatic spread by regulating the recruitment of myeloid cells and MDSCs in lung and regulating the release of IL10 and TGF-β with subsequent suppression of effector CD8 and CD4 T lymphocytes and induction of Treg generation (122) in a breast cancer preclinical model. Moreover, in absence of tumor specific T cells, the anaphylatoxin C5a promotes tumor growth by recruiting and activating myeloid-derived suppressor cells to release NO and ROS (123, 124). However, it was recently demonstrated that C3a and C5a have a pleiotropic and context specific role in tumor progression. Indeed the activation of the complement on tumor endothelium abrogates tumor endothelial barrier and restores T cell infiltration in tumor bed, especially in the presence of a tumor specific T cell response, improving thus adoptive T cell therapy efficacy (125, 126).

### MDSCs Favor Tumor Cells Intravasation Into Circulation

Following migration through the ECM, cancer cells should intravasate in the blood or lymphatic circulation. Therefore, within the primary tumor the promotion of new vessels formation appears as a key point for tumor cells dissemination. MDSCs can participate to this process inducing the development of a dysfunctional vasculature that is more permissive to tumor cell intravasation, as we will discuss later. Moreover, through the release of proteolytic enzymes such as MMP2 and MMP9, MDSCs can remodel the basal membrane, opening a route for neoplastic cell migration (127). For instance, tumor activated PMNs, recruited through HMGB1 produced by UV-damaged epidermal keratinocytes, promote cancer cell transmigration and enhanced metastasis (128).

However, the metastatic potential of CTCs depends on their ability to extravasate and colonize distant organs. In both melanoma and sarcoma models, tumor cells are trapped in capillaries due to size-restriction; however, also in the absence of a physical barrier CTCs can stop forming active adhesions to the endothelium (129). The balance between pro- and anti-tumoral inflammation appears as a crucial step of this process. Although NK cells and macrophages are capable to mediate the clearance of CTCs, myeloid cells activated toward a pro-tumorigenic phenotype can promote cancer cell survival and favor their adherence to the endothelium, boosting extravasation (56). Indeed, in both melanoma and liver cancer, PMN-MDSC-like cells can increase tumor cell retention and transendothelial migration by integrin (MAC-1)/ICAM-1 interaction (130, 131). Finally, both neutrophils and inflammatory monocytes (iMos), that in cancer setting resemble MDSCs (132), can physically associate with cancer cells supporting their extravasation (133).

### MDSCs Protect CTCs in Circulation and Promote Their Extravasation

Following shedding from the primary site, tumor cells enter the blood stream where they encounter an unfavorable environment created by the mechanical and physical sheer forces present inside the vessels (134, 135). Once entered the blood, the CTCs have to face a second challenge: they must escape the immune surveillance. One option to avoid the fatal encounter is to generate clusters. CTC clusters have been detected both in tumor-bearing mice and in cancer patients, and even though they represent a minority (2–4%) of the entire CTC population, they have higher probability to generate metastases than “lonely” CTCs (136). The CTC clusters escape the immune surveillance by physically interacting between themselves (homotypic interaction) or with leukocytes (heterotopic interaction). In this context Szczerba et al. (137) demonstrated that almost 50% of breast cancer patients have detectable CTCs in the blood, and among them, a small subset (3.4%) was composed by CTCs coupled with leukocytes. Through a single cell transcriptomic profiling, the authors demonstrated in several breast cancer preclinical models and cancer patients that these clusters comprise neutrophils with a N2-like signature, resembling PMN-MDSCs expressing ARG1, chemokine (C-X-C motif) ligand 2 (CXCL2), CCL2, VEGFA and endowed with pro-tumoral activity. A compelling finding was that CTC-neutrophil clusters led to fast metastases and short survival in mice and their presence correlated with poor prognosis in cancer patients. These results implied that neutrophils-associated CTCs gain a more aggressive phenotype than their homotopic cluster counterparts, which is linked to and increased mutational burden mediated by PMN-MDSC-derived ROS, on one side, and to an increased proliferation conferred by neutrophil-derived IL-6 and IL-1β on the other one. At levels below the genotoxic effect, ROS act indeed as mitogenic factor through the activation of NRF2-ARE-Notch axis (138–141). Particularly, it was shown that melanoma and breast patient-derived CTCs co-cultured with PMN-MDSCs activate Notch signaling via the direct interaction between Notch1R, present on the surface of CTCs, and Jagged1/DLL (Notch1 ligands) expressed on PMN-MDSCs (142). Interestingly, the concomitant activation of Notch signaling and ROS (i.e., H_2_O_2_) synergizes in enhancing CTC proliferation, *in vitro*. Thus, PMN-MDSCs sustain CTC survival through the activation of ROS-NRF2-ARE axis and Notch signaling pathway. Several mechanisms have been proposed to be involved in PMN-MDSC-CTC cluster formation. Two of these were identified on ICAM-1, expressed in CTCs and binding β2-integrin on neutrophils, and on VCAM-1 (137). Thus, ICAM-1 and VCAM-1 could represent good candidates to interfere with CTC-PMN-MDSC cluster formation and could be exploited to prevent metastasis formation. However, the feasibility of this innovative targeting approach needs to be validated by extensive experimental data, since the ICAM/VCAM axis is essential for several physiological processes.

Even though CTCs exploit different strategies to survive in circulation, their metastatic potential relies on the ability to extravasate and reach new tissues. While cancer cells are physically restrained in small venules, the extravasation from big vessels requires an active process supported by immune cells. For instance, the neutrophil extracellular traps (NETs), released by PMN-MDSCs, clog CTCs to favor their adherence to the endothelium supporting their extravasation and invasion (143). Thus, we can envision that preventing NET formation could block CTC-PMN-MDSC cluster formation. While the formation of NET favors the arrest of CTCs and their physical interaction with endothelial cells, PMN-MDSC can also potentiate tumor cell extravasation by directly increasing vessel permeability through the release of pro-inflammatory factors (i.e., IL-1β, MMP8, MMP9) and VEGFA, respectively (144, 145). Obviously, a large number of studies are needed for the development of NET-targeting approaches to avoid possible side-effects such as a limit response against pathogens.

### MDSCs Role on Generating Pre-metastatic Niche

For the colonization of metastatic site by cancer cells, a specific permissive microenvironment, defined as pre-metastatic niche (pMN), should be pre-established in distant organ [as extensively reviewed in (146, 147)]. The idea that tumor extrinsic determinants are actively involved on the preparation of a supportive environment before CTCs coming, was firstly proved by R.N. Kaplan and colleagues in 2005. In this pioneering study, the authors demonstrated that the infiltration of VEGFR-expressing immature myeloid cells induces the transformation of healthy tissues to future metastatic sites since these immature myeloid cells reach the distal metastatic site before the arrival of cancer cells (148). In fact, tumor-bearing mice display an increased amount in periphery of Lin^−^Sca1^+^cKit^+^ immature proliferating cells, that resemble BM-resident HSPCs, suggesting that tumors promote a reduced BM homing compared to tumor-free mice. These circulating pro-metastatic cells express in their membrane surface high amount of α4β1 integrins (also defined VLA-4) that mediate their arrest into fibronectin-rich environment in which pMN will be set up (148). Interestingly, the impact of immature myeloid cells on pMN establishment has also been confirmed in human setting. Indeed, Karaca et al. demonstrate that VEGFR-expressing myeloid progenitors are able to colonize sentinel lymph nodes before the arrival of CTCs (149). These circulating immature cells differentiate in mature CD11b^+^ cells (both CD11b^+^Ly6G^+^ and CD11b^+^Ly6C^high^ cells resembling M- and PMN-MDSCs, respectively) in distal tissues, generating a “muffle and fertile” soil where cancer cells can growth and expand (148). Therefore, the final differentiation of myeloid progenitors in pMN-MDSCs occurs mainly at the periphery rather than in the BM (150). Several TDSFs have been reported to steer the accumulation and expansion of myeloid precursors in pMNs (151). As known G-CSF, GM-CSF and IL-6 strongly influence MDSC differentiation and can also be used for *in vitro* MDSC culture (15, 16). Besides the effect on MDSC differentiation, GM-CSF is proven to be crucial for MDSC recruitment and accumulation at the tumor site (15, 18) while its role on pMN-development seems to be model-dependent (152). In contrast, G-CSF is sufficient to trigger MDSC infiltration in the lung in order to establish pMN, in breast tumor-bearing mice (153). Indeed, G-CSF mobilizes bombina variegate (Bv8)-producing CD11b^+^Ly6G^+^Ly6C^+^ cells that actively sustain pMN generation (152). The myeloid precursors differentiation into functional immunosuppressive MDSCs during pMN generation was recapitulated *in vitro* using cancer-cell derived supernatants highlighting the key role of forms like tyrosine kinase 3 (FLT3)-ligand, produced by cancer cells on sustaining this myeloid cell conversion (150). A number of tumor-derived chemokines can drive MDSCs infiltration into healthy tissues and support pMNs formation. CCL2 produced both in the target organ and in tumor can promote M-MDSC (defined as CD11b^+^CD115^+^Ly6C^hi^ cells) recruitment to the metastatic niche (145). More importantly, interfering the accumulation of these cells using a specific CCL2-blocking antibody strategy, prevent metastases generation (145). Both cancer and stroma cells contribute in MDSC accumulation in a CCL2-dependent manner. Increased cancer-derived CCL2 secretion is often triggered by genetic aberrations and dysregulated transcriptional program; in fact, p53 deletion and subsequent Rb protein inactivation in mouse sarcoma models switch on CCL2 production (154). Likewise, ΔNp63 transcriptional factor, which is often up-regulated in cancer cells, could directly induce CCL2 and CCL22 expression and the following metastatic spread by myeloid cell accumulation (155). Besides cancer cells, also cancer-associated fibroblasts (CAFs), characterized by the expression of fibroblast activation protein (FAP-)α, release high amount of CCL2, leading to sustain MDSC infiltration in pMNs (156). Interestingly, in intrahepatic cholangiocarcinoma patients, high levels of FAP have been associated with a worse metastatic prognosis (156). In colorectal cancer mouse model, instead, high amounts of CXCL1 released by TAMs have been reported to attract CXCR2-expressing MDSCs to generate liver pMNs (157). Other studies reported additional chemokines promoting MDSC transport to pMNs, such as MCP-1 (monocyte chemoattractant protein 1) (158), CCL12 (159), CCL9 (160), CCL15 (161), and CXCL17 (162), although the source of these cytokines in pMNs remains often unclear. Nowadays there are accumulating evidences that pro-metastatic molecules can be transported not only as soluble factors, but also inside tumor-derived microvesicles such as TEXs (13, 163, 164). Indeed, TEXs expressing distinctive integrin patterns guide the organotropism of metastases by orchestrating metastatic distribution and favor the generation of pMN by fusing themselves with resident cells (165). By a preferential tissue distribution, TEXs transfer their cargos, containing proteins, genetic materials and metabolites, to reprogram and educate pMN resident cells. MicroRNA (miR)122-derived from breast carcinoma TEXs, by inhibiting glucose uptake in non-tumor resident pMN cells, promote brain metastases (166). Moreover, MIF (macrophage migration inhibitory factor)-loaded pancreatic TEXs, promoting macrophages recruitment into liver pMNs, exacerbate liver metastatic burden (167). Similarly, TEXs mediate the production of pro-inflammatory S100A8 and S100A9 by pMN-resident endothelial cells that favors the expression of serum amyloid A (SAA)3 able to recruit CD11b-expressing myeloid cells by a TLR4-dependent pathway (168). Importantly, MDSCs can also synthesize and secrete high amounts of S100A8/A9 dimers (169) and exosomes derived from Gr1^+^CD11b^+^ MDSCs are able to carry these proteins (170). These findings suggest that S100A8/A9 factors maintain an autocrine feedback loop that favors accumulation of MDSC in pMNs. Indeed, S100A8/A9 molecules are important players in metastases generation by favoring both recruitment and differentiation of several pMN-infiltrating myeloid cell subsets commonly defined as Mac-1^+^ myeloid cells among the MDSCs (168, 169). In agreement with this, S100A9-deficient mice showed a strong impairment of MDSCs accumulation in liver and lung pMNs during colon metastatization (171). Exosomes could also transport signaling molecules from MDSCs to the other components of pMN, but this crosstalk is poorly characterized and needs further investigation.

In order to become available for colonization by CTCs, distant pMNs undergo several tissue alterations such as the generation of new blood vessels that provide oxygen and nutrients to proliferating cancer cells (146, 147). This process is termed 'angiogenic switch' and, in general, it is promoted in response to hypoxia (172). MDSCs play a critical role on initiating and sustaining the development of a new vascularization in pMN, primarily by secreting a variety of regulatory molecules such as VEGFA (173). Recently, Hsu et al. demonstrated that high amount of platelet-derived growth factor BB (PDGF-BB) released by pMN-infiltrating MDSCs increases angiogenesis and chaperone tumor cells through the bloodstream to new sites of metastasis (162). Another MDSC-associated proangiogenic factor is Bv8, which is released by a STAT3-dependent pathway (174, 175). The pro-tumor impact of Bv8-expressing MDSCs is confirmed by the high amount of these cells in tumor-bearing hosts undergoing refractoriness to anti-VEGF therapy (176). Similarly, MDSCs mediate also resistance to the antiangiogenic sunitinib, a tyrosine kinase inhibitor, both in preclinical (177) and clinical (178) settings of renal cell carcinoma. pMN-infiltrating MDSCs sustain angiogenesis also by producing high levels of MMP9 that promotes bioavailability of VEGF. Indeed, the genetic ablation of *Mmp9* restricts metastasis formation by normalizing the aberrant vasculature in pMNs (179). Interestingly, liver metastases-infiltrating MDSCs induce also the down-regulation of the antiangiogenic factor angiopoietin-like 7 (ANGPTL7) in cancer cells (180). During pMN establishment, MDSCs can also acquire some unexpected properties and features. Indeed, several studies reported the presence of an alternative MDSC subtype termed fibrocytes in patients with metastases (181, 182). Ou et al. demonstrated that fibrocytes can be generated in mouse cancer models from CD11b^+^Ly6G^+^ MDSC subset following a Kruppel-like factor 4 (KLF4)-dependent signaling (183). Moreover, MDSCs can undergo osteoclast differentiation and contribute to enhanced bone destruction and tumor growth in both breast cancer and myeloma models (184–186). Nowadays, the main knowledge about the role of MDSCs in pMN generation is derived from different mouse models in which cancer-cell derived factors, that support MDSC recruitment to pMN, have been studied broadly. Therefore, both the genetic and the epigenetic MDSC-reprogramming as well as the definition of key MDSC-associated properties during pMN development need to be deeply elucidated.

### MDSCs Involvement During Metastases Formation

Since most metastases present epithelial but not mesenchymal features, probably the EMT process is a temporary occurrence, and tumor cells, after seeding in pMN, revert their phenotype. This process is termed mesenchymal-to-epithelial transition (MET). MDSCs are actively involved in this process. In fact, in lung pMNs of MMTV-PyMT spontaneous breast cancer model, MDSCs secrete versican, an extracellular matrix chondroitin sulfate proteoglycan, that sustain MET process by reducing Smad phosphorylation in cancer cells (187). Interestingly, the frequency of versican-expressing intratumoral stromal cells correlates with a worse prognosis in women with node-negative breast cancer (188).

In contrast, MDSCs may also inhibit metastases. A single study reported that thrombospondin 1 (TSP-1)-expressing MDSC-like cells are able to abrogate the metastatic spread of prostate cancer cells (189) opening new insight on MDSC and metastasis relation. In the metastases framework, MDSC-associated immunosuppressive functions are regulated by oxidative stress and amino acid metabolism (8). MDSCs rely on fatty acid-β oxidation (FAO) to fuel the synthesis of inhibitory cytokines (i.e., IL-10, TGF-β) (190), which are generally required to both restrain T lymphocytes anti-tumor response and sustain tumor cell aggressiveness thus favoring metastases. In a recent publication (191), Hsu et al. demonstrated, in the 4T1 mouse breast cancer model, that the expression of *Csf3* by tumor cells is heterogeneous, with some 4T1 cells producing higher amounts (i.e., liver metastatic) than others (i.e., lung metastatic). CSF3 is functionally required for both maturation, proliferation and mobilization of neutrophils, and for the first time Hsu et al. demonstrated that among them, the low density neutrophils (LDNs), with characteristic of MDSC, are highly demanding for CSF3 in order to sustain their metabolic flexibility. In fact, while both normal density neutrophils (NDNs) and LDNs use glucose under nutrient supplements, LDNs are rapidly adapting to metabolic changes, like nutrient/glucose deprivation and hypoxia, and engage oxidative phosphorylation over glycolysis. Interestingly, the authors showed that pro-liver metastatic LDN-MDSC-like cells undertake mitochondrial metabolism to produce ATP, while NDNs use mitochondria to regulate apoptosis rather than producing ATP. Moreover, LDN-MDSC-like cells, were shown to perform NETosis to an extended degree than NDNs, using lipids as a source, in glucose deprived environments (i.e., metastatic liver). Indeed, LDNs were reported to have higher levels of lipids than their counterpart NDNs, providing fuel to the fatty acid oxidation pathway to sustain their high metabolic demand required for functional activity (mainly NETosis). Finally, under glucose and nutrient limitation, LDN-MSDC-like cells were shown to use glutamate and proline to induce NETosis. Interestingly, glutaminase, the enzyme involved in glutamate degradation, is stored inside the secondary granules, which are secreted upon NET induction. This could explain why LDN-MDSC-like cells prefer to convert glutamate to α-ketoglutarate to fuel the tricarboxylic acid (TCA), during glucose deprivation, rather than protein synthesis. In conclusion, pro-metastatic PMN-MDSCs are endowed with high metabolic flexibility to adapt to different microenvironments. This flexibility mainly resides in the use of lipids to carry out their functions, including NETosis and cytokine secretion.

## Concluding Remarks

Despite the extraordinary clinical achievements of immunotherapy on controlling metastatic diseases, our knowledge about molecular mechanisms and cell-networks that guide the metastatic process is still limited. Cancer cells are not an isolated and completely independent entity, but, in contrast, they act in concert with various cells in the body. By reprogramming myelopoiesis, cancer cells generate the “partners in crime,” like MDSCs. As described, MDSCs guide several aspects of tumor growth and metastatic cascade, such as cancer cell-stemness, immunosuppression, local invasion, angiogenesis, vasculogenesis, EMT/MET, CTC-protection and pMN formation; therefore, we can envision their use as targets to develop both innovative liquid biopsy-based cancer diagnostics as well as anti-cancer therapeutic approaches. To date MDSC-targeting approaches were preferentially validated to contrast primary tumor growth by acting on three main aspects of MDSC biology: MDSC trafficking and accumulation in primary tumor, MDSC functions and MDSC maturation/differentiation from BM precursors (Table 1). Basically, the abrogation of MDSC migration inside tumor are based on antagonist antibodies for specific chemokine receptors [i.e., CXCR2 (192); CCR5 (193)] or small molecules [i.e., CXCR4 (194)]; on the contrary, strategies targeting the immunosuppressive functions of MDSCs are based on specific pharmacological inhibitors abrogating the activity of transcriptional factors [i.e., STAT3 (31)] or key immunosuppressive-associated enzymes [i.e., COX-2 (82)], as well as on checkpoint inhibitors [i.e., PD-1L (85)]. Finally, various type of treatments, including conventional chemotherapy [i.e., Gemcitabine (38)], small molecules [i.e., sunitinib (195)] or biological agents [i.e., bevacizumab (196)] have been validated to limit the MDSC accumulation on tumor site or lymphoid organs. Interestingly, all these anti-MDSC treatments might be applied also to limit the metastatic process. In fact, the possibility to combine checkpoint-based immunotherapy with MDSC-targeting approaches may be the clinical standard goal in the near future to develop a personalized cancer therapy. The use of spontaneous metastatic mouse models able to recapitulate the biological features of the metastatic spread, the application of high throughput technologies able to deeply characterize the genetic, epigenetic and metabolic pathways as well as the identification of molecules that sustain the cross-talk between MDSCs and cancer cells, will clarify some unsolved aspects of the interaction between MDSCs and metastases and lay the groundwork to design more effective therapeutic strategies.

**Table 1:** Myeloid-derived suppressor cells targeting.

**Target**	**Class**	**Name**	**Clinical trial**
CCR5	Antibody, small molecules	Leronlimab, maraviroc, vicriviroc	NCT03631407; NCT03838367; NCT03631407
CCR5-Ig	Antibody		
CXCR2	Antibody		
CD21	Antibody		
CSF-R1	Small molecule	PLX647	
CXCR1/2	Small molecule	SX-682	NCT03161431
CXCR4	Small molecule		
STAT3	Different molecules	Naringenin (SOCS3); ruxolitinib (phosphorylation); STA-21 (dimerization); Stattic (phosphorylation); S31–201 (dimerization); AZD9150; MMPP (DNA binding); siRNA	NCT02417753;
PDE5	Small molecule	tadalafil	NCT02544880; NCT01697800
HDAC	Small molecule	Entinostat	NCT03250273
ARG1	Small molecule; vaccine	CB-1158; ARG1 peptides	NCT02903914; NCT03837509; NCT03689192
IDO	Small molecule	Epacadostat; BMS-986205	NCT04047706; NCT01961115
c-FLIP	Chemotherapy	5-FU	
PD-1	Antibody	Nivolumab, pembrolizumab	NCT03302247; NCT03161431; NCT03631407
PD-L1	Antibody	Durvalumab, atezolimumab	NCT02827344
Fatty acids	Small molecule	Etoxomir	
Protein nitration	Small molecule	Nitroaspirin	
COX-2	Small molecule	Celecoxib; SC58236, SC58125	NCT02432378
ROS scavengers	Small molecules	Synthetic triterpenoids	
NO donor	Small molecule	AT38	
TRAIL-R2	Antibody	DS-8273a	NCT02991196
MMP9	Small molecule		
Amino-bisphosponates	Small molecule	Zoledronate	
TK	Small molecule	Sunitinib; axitinib; imatinibe; nilotinib	NCT03214718
All-trans retinoic acid	Small molecule	Vesanoid	NCT02403778
Vitamin D3	Vitamin		
DNA	Chemotherapy	Docetaxel	
DNA	Chemotherapy	Gemcitabine	NCT03302247; NCT02538432
c-KIT	Small molecule	Imatinib	NCT00852566
VEGF-A	Antibody	Bevacizumab	NCT02669173; NCT02090101
histamine receptor 2 (H2)	Small molecule	Ranitidine; famotidine	NCT03145012

*The MDSCs recruitment (blue section), immunosuppression (green section) and maturation /differentiation (orange section) are altered by targeting specific molecules. Targets and corresponding class and name of inhibitors identified and tested both in vitro and preclinical studies are highlighted. The clinical trial codes, for some of the drugs being tested to target MDSCs in cancer patients, are indicated*.

## Author Contributions

All authors listed have made a substantial, direct and intellectual contribution to the work, and approved it for publication.

### Conflict of Interest

The authors declare that the research was conducted in the absence of any commercial or financial relationships that could be construed as a potential conflict of interest.
